# *Staphylococcus aureus* blocks host autophagy through circSyk/miR-5106/Sik3 axis to promote progression of bone infection

**DOI:** 10.1371/journal.ppat.1012896

**Published:** 2025-01-27

**Authors:** Zhihao Chen, Qiyuan Jin, Jinqi Zhong, Zonggang Xie, Qi Chen, Liubing Li, Jijie Li, Chenhao Zhao, Junfeng Wang, Xiaoying Tang, Mingxiao Han, Ru Li, Ziyuan Li, Zelei Tong, Min Wang, Hong Du, Haifang Zhang

**Affiliations:** 1 Department of Orthopedics, The Second Affiliated Hospital of Soochow University, Suzhou, China; 2 Department of Clinical Laboratory, The Second Affiliated Hospital of Soochow University, Suzhou, China; 3 Department of Clinical Laboratory, The Nuclear Industry 417 Hospital, Xi’an, China; 4 Department of Clinical Laboratory, Kunshan Municipal Third People’s Hospital, Suzhou, China; 5 MOE Key Laboratory of Geriatric Diseases and Immunology, Soochow University, Suzhou, China; Trinity College Dublin, IRELAND

## Abstract

With the rapid increase in the number of implant operations, the incidence of bone infections has increased. Methicillin-resistant *Staphylococcus aureus* (*S. aureus*) and other emerging fully drug-resistant strains make the management of bone infections even more challenging. Bone infections are mainly caused by *S. aureus* and require extensive surgical intervention and long-term antibiotic therapy. The host autophagy response is critical to the elimination of *S. aureus* infections. In this study, we demonstrate that a circular RNA (circRNA), circSyk, is a potential biological target for the treatment of *S. aureus*-induced bone infection. Most importantly, *S. aureus* regulates circSyk to block autophagy and promote bone destruction via the circSyk/miR-5106/Sik3 axis in a nonclassical pathway, which is involved in the *S. aureus* infection process through a competitive endogenous RNA network. In summary, this study proposes a novel perspective on the immune escape of *S. aureus* in bone infections, based on circRNA.

## Author summary

Bone infection is mainly caused by *Staphylococcus aureus*, and results in progressive inflammation and bone destruction, requiring extensive surgical interventions and long-term antibiotic treatment. However, evidence has shown that *S*. *aureus* can survive inside a variety of cells, including osteoclasts, which is considered to underlie the persistent bone infections caused by *S*. *aureus.* Circular RNAs are noncoding RNAs that play important roles in bone infection. Following from our previous transcriptomic analysis of circRNA expression in response to the infection of osteoclasts by *S. aureus*, we show that *S. aureus* upregulates circSyk to block osteoclast autophagy and promote bone destruction via the circSyk/miR-5106/Sik3 axis.

## Introduction

With the rapid increase in the number of implant operations, including joint replacement, spinal surgery, and dentistry, combating bone infections has become extremely important [1–4]. Bone infections are most commonly caused by *S. aureus*, and involve progressive inflammation and bone destruction, requiring extensive surgical intervention and long-term antibiotic treatment [5]. Bone infection can be catastrophic for patients, with significant morbidity and even mortality arising from implant failure, disease relapse, and even amputation or severe sepsis [6,7]. Methicillin-resistant *S. aureus* and other newly emerging fully antibiotic-resistant strains complicate its treatment and clinical outcomes [8]. *S. aureus* is a facultative intracellular pathogen, which can evade host immune defense [9–11] and most therapeutic drugs through proliferating within cells [8]. Therefore, nonantibiotic interventions are urgently required to control these refractory bone infections.

Autophagy plays a vital role in the host immune response to bacteria [12]. In the classical pathway, the host autophagic response is critical to the elimination of *S. aureus* infection [13,14]. Gibson *et al.* reported that the host protein associated with autophagy, p62, limits *S. aureus* survival by regulating neutrophil phagocytosis, which significantly affects the host immune defense and susceptibility to *S. aureus* [15]. It has also been reported that host ATG16L1 regulates autophagy to limit the damage caused by bacterial α-toxin, increasing the cellular survival rate during *S. aureus* infection [16]. However, there is a noncanonical pathway through which *S. aureus* inhibits host autophagy to maintain its infection, as reported by Mulcahy *et al*., who demonstrated that *S. aureus* subverts autophagy in polymorphonuclear neutrophils to maintain intracellular infection [17]. In the study of Cai *et al*., *S. aureus* was shown to evade autophagic degradation by blocking autophagic flux and increasing the pH of autophagolysosomes in macrophages [18].

Circular RNAs (circRNAs) are highly conserved small noncoding RNA molecules with a closed circular structure [19]. The competitive endogenous RNA (ceRNA) network, often referred to as the ‘sponge mechanism’, is regarded as the primary way in which circular RNAs (circRNAs) exert their significant biological functions [20,21]. This ‘sponge mechanism’ involves circRNAs binding to microRNAs (miRNAs), thereby sequestering and preventing the inhibition of target genes by miRNAs, much like a sponge absorbs water, whereas circRNAs can also act as protein inhibitors [22]. Previous studies have shown that circRNAs are widely involved in the immune response to bacterial infection [23–25]. Huang *et al*. reported that circRNA-0003528 promoted tuberculosis-related macrophage polarization by stimulating CTLA4 expression through miR-224-5p, miR-324-5p, and miR-488-5p [26]. Zhang *et al.* showed that circRNA-1806 reduces T-cell apoptosis and G1S arrest in T cells by sequestering miRNA-126 and increases bacterial survival rate during cryptococcal infection [27]. Liu *et al.* reported that circDNMT3B regulates d-lactic acid, malondialdehyde, superoxide dismutase activity, and interleukin 6 and interleukin 10 levels through miR-20b-5p to reduce intestinal mucosal permeability dysfunction, and therefore has therapeutic potential for sepsis [28]. However, the roles of host circRNAs in infections caused by *S. aureus* are still unclear.

The pathway by which *S. aureus* evades the host’s immune response is considered to be associated with repeated persistent bone infections. In our previous study, we provided the first global transcriptomic profile analysis of circRNAs in osteoclasts infected by intracellular *S*. *aureus*, and showed that circRNAs may play an important role in *S. aureus*-induced bone infection [29]. In the present study, we demonstrate that circSyk is a potential biological target for the treatment of *S. aureus*-induced bone infection. Most importantly, in a nonclassical pathway, *S. aureus* induces circSyk to block autophagy and promote bone destruction via the circSyk/miR-5106/Sik3 axis. The circSyk/miR-5106/Sik3 axis is involved in the *S. aureus* infection process through a ceRNA-based mechanism. Considering the increasingly severe challenges posed by bone infection, we believe that our research demonstrates an important mechanism of *S. aureus* immune escape during bone infection, and provides a basis for the future development of diagnostic, treatment, and drug design strategies.

## Materials and methods

### Ethics statement

Ethical approval was obtained from the Ethics Committee of the Second Affiliated Hospital of Soochow University and the approval number is LK2024002. The formal consent was written by all the patients and healthy individuals participated in this study.

### Culture of RAW 264.7 cells and bacterial strains

Mouse mononuclear macrophage leukemia (RAW 264.7) cells, human embryonic kidney (293T) cells, and *S*. *aureus* (ATCC 25923) are maintained in the laboratory of the Second Affiliated Hospital of Soochow University. RAW 264.7 cells and 293T cells were cultured in Dulbecco’s modified Eagle’s medium (HyClone, USA) containing 10% fetal bovine serum (FBS) at 37°C under 5% CO_2_. To culture bacteria, *S*. *aureus* colonies were selected and cultured overnight in 20 mL of Luria–Bertani (LB) isotonic liquid medium at 37°C with shaking at 250 rpm. The bacterial LB suspension was then added to 20 mL of novel LB isotonic liquid in a ratio of 1:100, and shaken at 250 rpm and 37°C until an optical density at a wavelength of 600 nm (OD_600_) of 0.6 was reached.

### Induction of osteoclasts and infection with *S*. *aureus*

RAW 264.7 cells were seeded in a six-well plate at 2.5 × 10^5^ cells/well in α-Minimal Essential Medium (HyClone) containing 10% Fetal Bovine Serum (FBS), 100 ng/mL soluble RANKL (R&D Systems, USA), 0.002 mol/L l-glutamine, 100 units/mL penicillin, and 100 µg/mL streptomycin. The cell culture medium was changed every second day and multinucleated osteoclast formation was observed under the microscope after a 5-day induction period in culture, as reported previously [29,30]. Osteoclasts were infected with prepared *S*. *aureus* in a ratio of 1:10. Following incubation at 37°C for 2 h, the cells were incubated with 100 µg/mL gentamicin for 1 h to kill any extracellular bacteria. Osteoclasts treated with the same volume of sterile phosphate-buffered saline (PBS) were used as the control group.

### Verification of circSyk in patients’ serum samples

Sixteen serum samples were obtained from patients with *S. aureus*-induced bone infection, and sixteen serum samples from healthy individuals were used as the control. Total RNA was extracted from the serum samples with TRIzol Reagent (Invitrogen, USA) and treated with the HiScript III 1st Strand cDNA Synthesis Kit (Vazyme, China) to generate cDNA. Reverse Transcription Quantitative PCR (RT-qPCR) was performed on the Applied Biosystems StepOnePlus Real-Time PCR System (Thermo Fisher Scientific, USA) with AceQ qPCR SYBR Green Master Mix (Vazyme, China). GraphPad Prism v7.0 (GraphPad Software, La Jolla, CA, USA) was used for the receiver operating characteristic (ROC) curve analysis of circSyk in patients with *S. aureus*-induced bone infection and healthy individuals.

### Small interfering RNA (siRNA) and miR-5106 mimics

The circSyk siRNA (siRNA–circSyk), miR-5106 mimics and negative control siRNA (siRNA–NC) were provided by GeneChem Co., Ltd (Shanghai, China), and the sequences were shown in S1 Table. Negative control siRNA, which shares no homology with any known mammalian gene, was provided by GeneChem Co., Ltd (Shanghai, China). Lipofectamine 2000 (Invitrogen) was used for transfection, according to the manufacturer’s instructions. Lipofectamine 2000 was adapted to transfect siRNA–circSyk, miR-5106 mimics and siRNA–NC, after 5 days of osteoclast induction.

### Immunofluorescence

Osteoclasts were cultured on coverslips and achieved to 70–80% confluency. The cells were initially grouped and pre-treated according to the specific objectives of the experiment and then infected with *S. aureus* for 2 h to allow internalization of *S. aureus*. Following treatment, the cells were rinsed with PBS and fixed on slides with 4% paraformaldehyde for 30 min, followed by additional PBS washes. The cells were permeabilized with PBS containing 0.25% Triton X-100 for 10 min at room temperature. After rinsing with PBS, the cells were incubated with a goat serum blocking solution for 30 min, followed by primary antibodies diluted in blocking solution at 4°C overnight. Then, the coverslips were incubated with fluorochrome-conjugated secondary antibody and Phalloidin-iFluor for 1.5h. Following PBS washes, the coverslips were mounted on glass slides using an antifade mounting medium to preserve fluorescence. The primary antibody was as follows: rabbit anti-*Staphylococcus aureus* (Abcam, ab20920, 1:1000). The antibodies was as followsThe second antibodies were as follows:goat anti-rabbit IgG Alexa Fluor 488 (Abcam, ab150077, 1:500), Phalloidin-iFluor 647 (Abcam, ab176759). Images were captured on a Zeiss LSM 900 confocal laser scanning microscope. The fluorescence intensity was analysed with ImageJ 1.53t software. Moreover, the cell lysates obtained by osmotic shock were serially diluted and plated onto LB agar plates, followed by incubation at 37°C overnight to quantify the intracellular *S. aureus* as described previously [31].

### Autophagy double-labeled adenovirus (mCherry-GFP-LC3B) transfection

The Ad-mCherry-GFP-LC3B adenovirus was purchased from Beyotime (C3011-1ml). Osteoclasts were seeded on coverslips and diluted mCherry-GFP-LC3B adenovirus (10 MOI) was added to the cells and incubated under conditions of 37°C and 5% CO_2_ for 6 hours. Following incubation, the virus-containing medium was aspirated and replaced with fresh complete DMEM medium. Cells were then subjected to various treatments according to the specific objectives of the experiment. When mCherry is used to colabel LC3B with GFP, the acidic environment within lysosomes leads to the quenching of GFP fluorescence during the fusion of autophagosomes with lysosomes, while the stable mCherry fluorescence is retained in the merged image. In microscopic imaging, red dots represent autolysosomes, and the overlap of red and green fluorescence appears as yellow dots, indicating autophagosomes. The number of red and yellow spots can be used to indicate the level of autophagy.

### Reverse transcription quantitative polymerase chain reaction (RT-qPCR)

Specific primers for each target sequence were designed based on the sequences of linear transcripts. Total RNA was extracted from osteoclasts and serum samples with TRIzol Reagent (Invitrogen) and treated with RNase-free DNase I (Vazyme, China) to eliminate any traces of contaminating DNA. RT-qPCR was performed on the Applied Biosystems StepOnePlus Real-Time PCR System (Thermo Fisher Scientific) using qPCR SYBR Green Master Mix (Vazyme, China). The expression levels of circRNAs and mRNAs detected with RT-qPCR were all normalized to that of the endogenous control gene *GAPDH* with the 2^−ΔΔCT^ method. The expression levels of miRNAs detected with RT-qPCR were normalized to that of the endogenous control gene *U6* with the 2^−ΔΔCT^ method. All primer sequences are shown in S2 Table.

### Luciferase reporter assay

The circSyk-binding sites of miRNA were predicted with microT-CDS v5.0, TarBase v7.0, and TargetScan. The binding sites of miR-5106 and *sik3* 3’UTR were predicted wtih TargetScan. According to the method reported previously[32,33], luciferase assay was performed using 293T cells transfected with the GP-miRGLO plasmid (GenePharma, China) containing different fragment sequences (wild-type and mutant-type). Their sequences were provided in S1 Table. Following transfection, cells were incubated for 48 hours and then treated with miR-5106 mimics or siRNA-NC as control. Luciferase activity was measured using a microplate luminometer after adding the luciferase substrate provided in the Dual-Luciferase Assay Kit (Vazyme, China), and results were normalized to Renilla luciferase. Data were analyzed using appropriate statistical methods.

### Western blotting

Total osteoclast proteins were extracted with radio immunoprecipitation assay (RIPA) lysis buffer (Beyotime, China) containing protease and phosphatase inhibitor cocktail (Beyotime, China). The protein concentrations were determined with the Bicinchoninic Acid Assay (BCA) Protein Assay kit (Beyotime, China). The proteins were separated with SDS-PAGE and transferred to polyvinylidene difluoride membranes (Merck Millipore, Germany), which were blocked with 5% nonfat milk and incubated with primary antibody directed against SIK3 (diluted 1:1000; Biorbyt, UK), LC3B (1:1000; Cell Signaling Technology, USA), p62 (diluted 1:1000; Cell Signaling Technology), or β-actin (diluted 1:1000; Cell Signaling Technology) at 4°C overnight. The membranes were then washed with Tris-buffered saline containing Tween 20 and incubated with horseradish-peroxidase-conjugated secondary antibody for 1 h at room temperature. The protein bands were visualized with the enhanced chemiluminescence method by Enhanced Chemiluminescence reagent (Millipore, USA). β-Actin was used as the loading control.

### Murine model of bone infection

We used a transtibial implant mouse model to assess *S. aureus* infections, as previously described [29,34,35]. In brief, 6–8-week-old C57BL/6 mice were anesthetized with ketamine (100 mg/kg) and xylazine (10 mg/kg). A flat stainless steel wire (MicroDyne Technologies, USA) with a cross-section of 0.2 mm × 0.5 mm was cut to 4 mm length and bent into an L-shaped implant: long side 3 mm, short side 1 mm. After sterilization, the implants were submerged in a culture of *S. aureus* (ATCC 25923) at 5 × 10^5^ colony-forming units (CFU)/mL for 30 min, and was implanted into the tibiae of female C57BL/6 mice. In the control group, mice were implanted with sterile pins. At 12 days, the tibiae of the mice in both groups were collected.

### Adenovirus construction

In the mice model of bone infection, to knock down circSyk, siRNA–circSyk and siRNA–NC were each cloned separately into the pLVX-IRES Puro vector and adenoviruses were constructed by GeneChem Co., Ltd. An miR-5106 miRNA mimic was also constructed by GeneChem Co., Ltd. The mice in the model of bone infection were injected intravenously via the tail vein with 100 μL of adenovirus expressing siRNA-circSyk or siRNA-NC 2 weeks before they were inoculated with *S. aureus* or sterile PBS.

### Transmission electron microscopy

The cell and tibiae samples from the mouse model of *S. aureus*-infected bone were collected, fixed with 2.5% glutaraldehyde, and osmificated with 1% aqueous osmium for 1 h at room temperature. The samples were then dehydrated with a graded series of acetone and embedded in Spurr’s epoxy resin. Thin sections were cut with a diamond knife, placed on formvar-carbon-coated nickel slot grids, and examined under a Zeiss TEM910 transmission electron microscope (Carl Zeiss, Germany).

### Bacterial load assay

Tibia related tissue including tibia, tibial implant, the surroundinge abscesses, and main organ were collected and and stored in 1 mL of room temperature sterile PBS. Tissues or organs were homogenized (RWD Life science, China) in 1mL of PBS. The implant was sonicated for 2 min to dislodge attached bacteria, which were collected with tibia related homogenates. Next, the homogenates were serially diluted, plated on blood agar (BA) plates, and incubated overnight at 37°C. To confirm *S. aureus* on the plates, random colonies were selected, and Staph Latex agglutination test (KERONG, China) was performed. Bacterial colonies were enumerated, and the generated CFU data were presented as CFUs per gram of tissue.

### Histological analysis with hematoxylin and eosin (H&E)

For histological analysis, tibia samples were fixed with formalin and then decalcified in 10% EDTA with continuous shaking for 3 weeks. Next, they were embedded in paraffin. After that, the samples were further sectioned into 4 µm-thick for staining. The sections were stained with hematoxylin for 5 min and eosin for 2 min at 22–24 °C and then visualized and photographed under a high-resolution microscope (Carl Zeiss, Germany).

### Microcomputed tomography (microCT)

After the bones were cleaned of all muscle and soft tissue, they were assessed with a 1076 CT scanner (SkyScan, Aartselaar, Belgium) using an X-ray source of 70 kV, 100 mA, and a pixel size of 18 μm. The images were reconstructed with the SkyScan NRecon software. Regions of newly generated bone were detected with the CT Analyzer software. The microarchitectural parameters of the volume of cortical bone destroyed and the bone volume (BV) fraction were assessed in the areas of the femurs with defects. The trabecular number (Tb.N), bone mineral density (BMD), and bone volume fraction (BV/TV) were used to measure the changes in bone quality.

### Statistical analysis

The statistical software GraphPad Prism v7.0 (GraphPad Software) was used for all statistical analyses. Quantitative data are expressed as means ± standard deviations. The statistical significance of differences between two groups was analyzed with Student’s *t* test. The criteria used to define differentially expressed circRNAs (DEcircRNAs) and differentially expressed mRNAs (DEmRNAs) with RNA-seq were: fold change ≥ 2.0 (*P* < 0.05) and false discovery rate < 0.05. The criterion used to confirm the downstream targets of DEcircRNAs and DEmiRNAs identified with RT-qPCR was: fold change ≥ 2.0 (*P* < 0.05). Correlation between two variations was analyzed with Pearson’s linear correlation analysis. The miRanda and TargetScan databases were used to predict the downstream targets of circRNAs and miRNAs, respectively [36,37]. *P* < 0.05 was considered to indicate statistical significance.

## Results

### Verification of the structure and clinical features of circSyk

**Fig 1:**
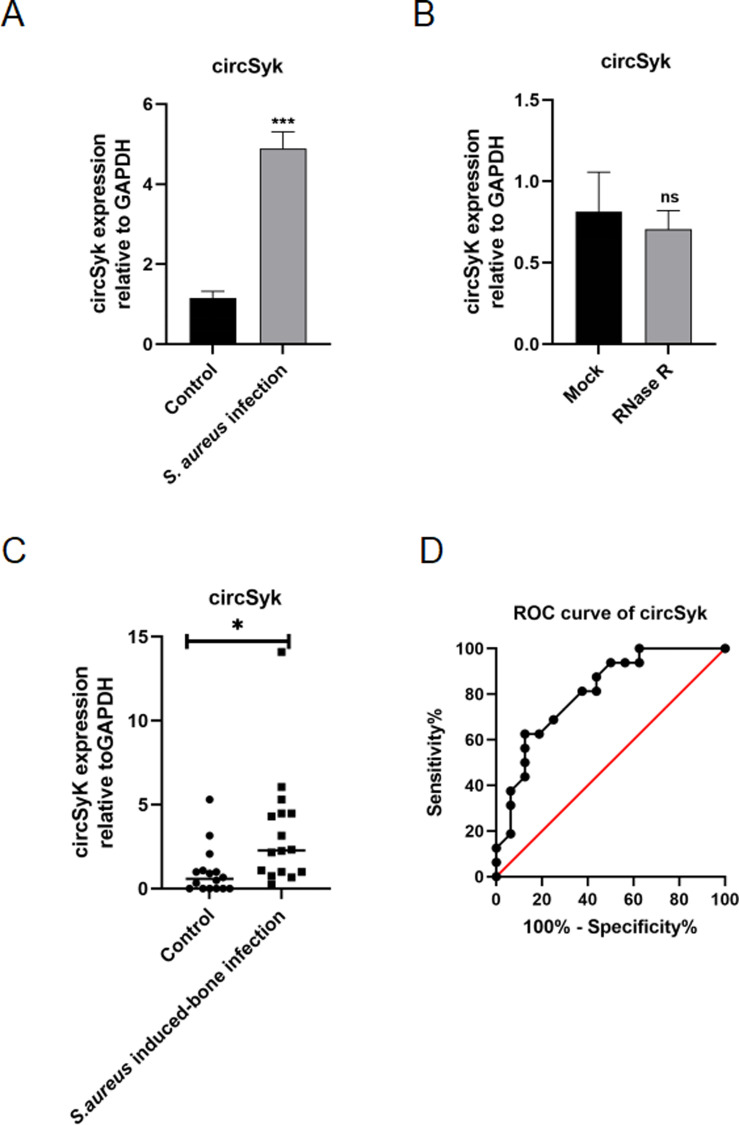
Verification of circSyk structure and clinical features. (A) circSyk expression was analyzed with RT-qPCR. Osteoclasts treated with the same volume of PBS were used as the control. (B) circSyk expression in osteoclasts treated with RNase R. Osteoclasts treated with the same volume of RNase-free water were used as the control (Mock). (C) circSyk expression in serum samples from patients with *S. aureus*-infected bone was evaluated with RT-qPCR (n=16). Serum samples from healthy individuals were used as the control (n=16). (D) ROC curve analysis of circSyk in patients with *S. aureus*-infected bone. Data are means ± SD of three independent experiments (*P < 0.05; ***P < 0.001; ns, nonsignificant).

In our previous study, DEcircRNAs were identified in *S*. *aureus*-infected osteoclasts [29]. In the present study, among these DEcircRNAs, circSyk showed significantly elevated expression in *S. aureus-*infected osteoclasts relative to the control group by employing the established *S. aureus*-osteoclast infection model (Fig 1A). Then, we examined the structural features of circSyk. Ribonuclease R (RNase R) digests almost all linear RNAs, but not circRNAs [38]. Therefore, we pretreated all RNAs with RNase R before RT-qPCR to verify their cyclic structure. Compared with the control group treated with RNase-free water, circSyk showed resistance to RNase R, indicating that circSyk has a stable ring structure (Fig 1B).

Based on the structural characteristics of circSyk, we further explored its clinical relevance in patients with *S. aureus*-caused bone infections. Sixteen serum samples from patients with *S. aureus*-caused bone infection and sixteen serum samples from healthy individuals were obtained, and the clinical characteristics of patients with *S. aureus*-caused bone infection were shown in S3 Table. Compared with the healthy individuals, circSyk expression was significantly elevated in the sera of patients with *S. aureus*-infected bone (Fig 1C). Moreover, a ROC curve analysis showed that circSyk is a promising biological target for the treatment of *S. aureus*-infected bone. The area under the curve (AUC) was 0.81. The sensitivity and specificity of circSyk in the detection of *S. aureus*-infected bone were 81.25% and 62.50%, respectively (Fig 1D).

### Downstream miRNAs of circSyk

**Fig 2:**
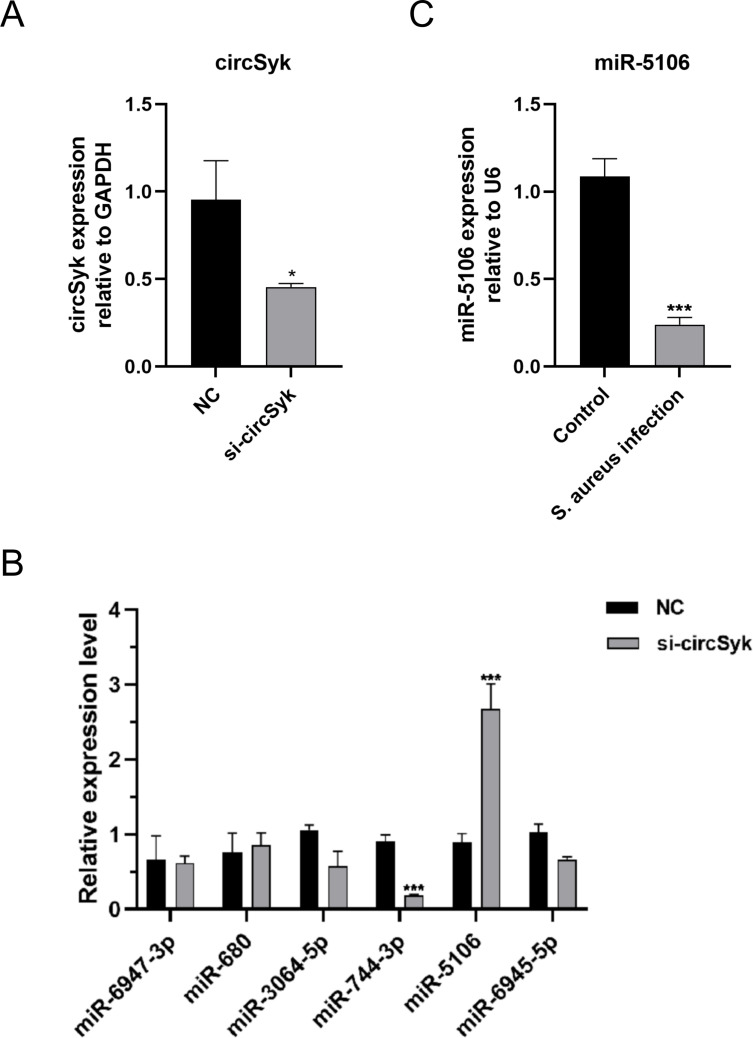
Identification of miRNAs downstream from circSyk. (A) Validation of circSyk knockdown. Osteoclasts transfected with the negative control siRNA (siRNA-NC) were used as the control (NC group). (B) Expression of miRNAs downstream from circSyk. Expression of miR-5106 was significantly downregulated in siRNA-circSyk group relative to NC group. Osteoclasts transfected with siRNA-NC were used as the NC group. (C) Validation of miR-5106 expression in osteoclasts with *S. aureus* infection. Osteoclasts treated with the same volume of PBS were used as the control. Data are means ± SD of three independent experiments (*P < 0.05; ***P < 0.001; ns, nonsignificant).

To study the mechanism of circSyk function in bone infection, we analyzed a public database to identify miRNAs associated with circSyk. We screened the miRanda database for miRNAs associated with circSyk, and 35 miRNAs were identified (S4 Table). We first ranked the predicted the targeted miRNAs based on their free energy, and the top six miRNAs (mmu-miR-5106, mmu-miR-6945-5p, mmu-miR-680, mmu-miR-6947-3p, mmu-miR-3064-5p, and mmu-miR-744-3p) were considered candidate miRNAs downstream of circSyk. Next, we effectively knocked down the expression of circSyk by siRNA-circSyk and the expression of circSyk clearly decreased in siRNA-circSyk-transfected osteoclasts as shown in Fig 2A.

Based on ceRNA mechanism, the downstream miRNA would be upregulated with transfection of siRNA-circSyk. So, we detected the expression changes of candidate miRNAs in siRNA-circSyk-transfected osteoclasts by RT-qPCR. NC-transfected osteoclasts were considered as control. The fold change ≥ 2.0 (P < 0.05) was employed as selected criterion. We found that only the expression level of miR-5106 was significantly upregulated in the siRNA-circSyk-transfected osteoclasts (Fig 2B). Although miR-744-3p showed significant downregulation in the si-circSyk group, this was not consistent with ceRNA mechanism.

In addition, we investigated the expression changes of miR-5106 in the *S. aureus*-infected osteoclasts and uninfected control osteoclasts. As shown in Fig 2C, the expression of miR-5106 in *S. aureus*-infected osteoclasts was downregulated significantly. Additionally, although the other five miRNAs were downregulated, their decrease was not nearly as pronounced as that of miR-5106 (S1A Fig). Consequently, miR-5106 was considered a potential downstream target of circSyk in *S. aureus*-infected osteoclasts.

### Target mRNAs of miR-5106

**Fig 3:**
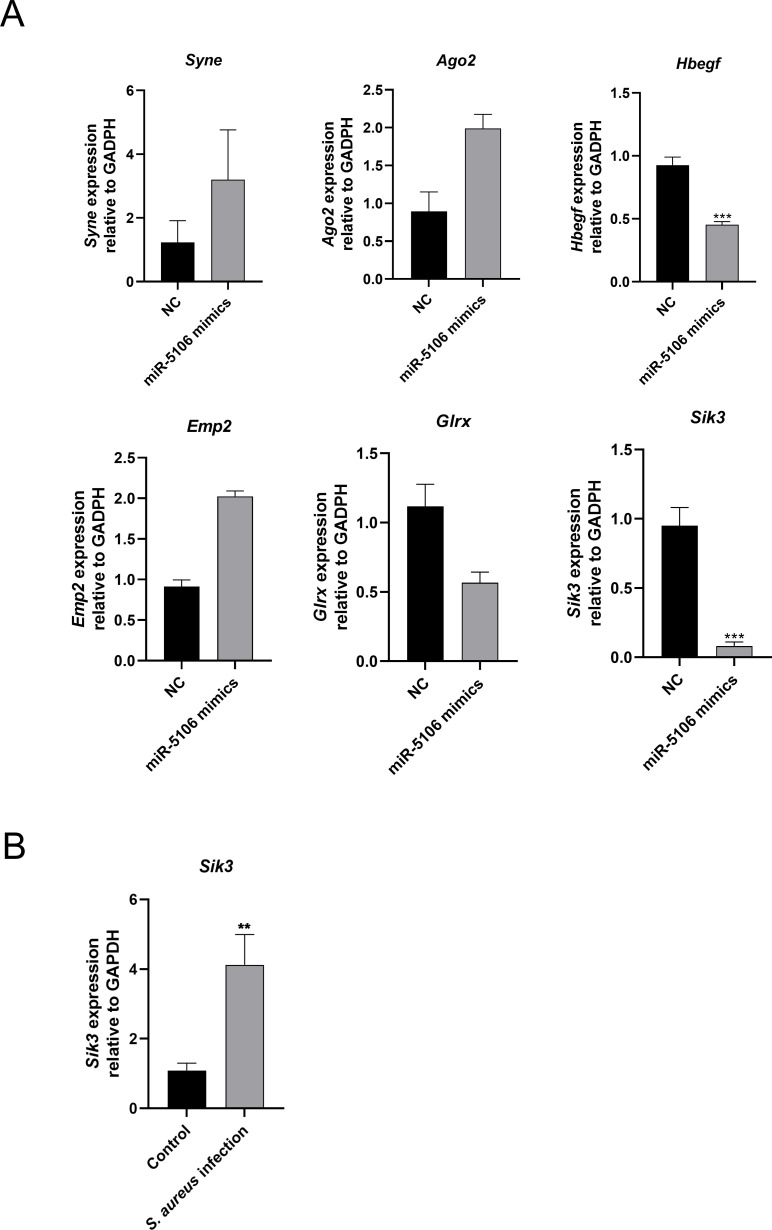
Identification of mRNAs downstream from miR-5106. (A) Expression of mRNAs downstream from miR-5106. Osteoclasts transfected with NC-mimics were used as the negative control. (B) Validation of *Sik3* expression in osteoclasts with *S. aureus* infection. Osteoclasts treated with the same volume of sterile PBS were used as the control. Data are means ± SD of three independent experiments (**P < 0.01; ***P < 0.001; ns, nonsignificant).

Based on high-throughput RNA-seq expression profiling reported previously, we identified the DEmRNAs in RAW 264.7-induced osteoclasts with *S. aureus* infection (GSE161629) [29]. The TargetScan database was used to construct a target network between these six miRNAs and their potential target mRNAs. In total, 2167 mRNAs were identified with a total context++ score of <−0.1 in the TargetScan database. In GSE161629, 334 DEmRNAs were identified with a fold change ≥ 2.0 (*P* < 0.05) and a false discovery rate < 0.05. When the mRNAs from GSE161629 were compared with those in the TargetScan database, six mRNAs were identified (*Ago2*, *Emp2*, *Glrx*, *Hbegf*, *Syne*, and *Sik3*) for further validation. Those mRNAs with fold change ≥ 2.0 (*P* < 0.05) were considered candidate downstream targets of miR-5106. We then transfected Raw-264.7-induced osteoclasts with an miR-5106 mimic, which greatly reduced *Sik3* expression levels comparing with NC group (Fig 3A). The expression of *Hbegf* was also decreased significantly, but its fold change < 2.0. However, in Raw264.7-induced osteoclasts infected with *S. aureus*, Sik3 expression was significantly upregulated (Fig 3B). These data suggest that circSyk, miR-5106, and *Sik3* may form a circRNA/miRNA/mRNA axis to modulate the biological function of osteoclasts after *S*. *aureus* infection.

### CircSyk regulates Sik3 expression through miR-5106 in *S. aureus*-infected osteoclasts

**Fig 4:**
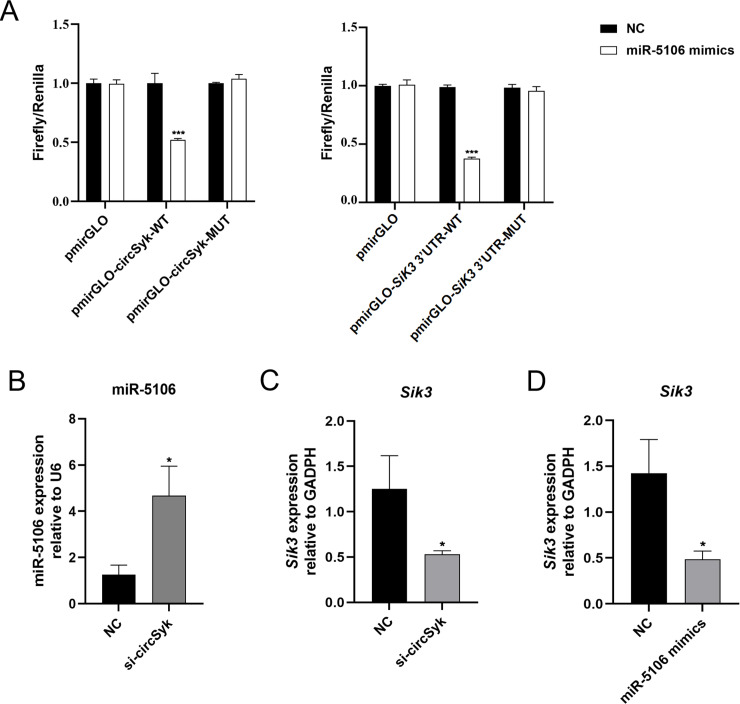
circSyk regulates *Sik3* expression directly through miR-5106 during *S. aureus* bone infection. (A) Dual-luciferase assay of the binding sites between circSyk and miR-5106, miR-5106 and sik3. 293T cells transfected with NC-mimics was used as the control (NC group) for the miR-5106 mimics group. Luciferase reporter constructs containing either a WT *SiK3* 3’UTR (3’ UTR-WT) or this same region after site-directed mutagenesis (3’UTR-MUT). Firefly luciferase activity was quantified and normalized to Renilla luciferase activity as the internal reference. (B) Expression of miR-5106 determined with RT-qPCR. *S.aureus*-infected osteoclasts were transfected with siRNA-circSyk or siRNA-NC. NC group, the control group. (C) Expression of *Sik3* determined with RT-qPCR In *S.aureus*-infected osteoclasts transfected with siRNA-circSyk or siRNA-NC. NC group, the control group. (D) Expression of *Sik3* was determined with RT-qPCR in *S.aureus*-infected osteoclasts transfected with miR-5106 or NC-mimics. NC group, the control group. Data are means ± SD of three independent experiments (*P < 0.05; ***P < 0.001; ns, nonsignificant).

To confirm the existence of the circRNA/miRNA/mRNA axis, we performed a luciferase reporter experiment, as previously described [32,33]. As shown in Fig 4A, the luciferase activity from the wild-type vector was significantly reduced in the group of miR-5106 mimics. These results confirmed that circSyk binds miR-5106 and miR-5106 binds 3’UTR of *Sik3* directly as well.

After the osteoclasts transfected with siRNA-circSyk or siRNA-NC were infected by *S. aureus*, the expression of miR-5106 was significantly higher (Fig 4B) and the expression of *Sik3* was significantly lower in the siRNA-circSyk-transfected group than the NC group (Fig 4C). Moreover, with *S. aureus* infection, the expression of *Sik3* was significantly downregulated in the osteoclasts transfected with the miR-5106 mimic (Fig 4D). These results collectively underscored the regulatory pathway of circSyk/miR-5106/Sik3 axis in osteoclast infected by *S. aureus*.

### Impact of the circSyk/miR-5106/Sik3 axis on autophagy and intracellular survival of *S. aureus* in osteoclasts

**Fig 5:**
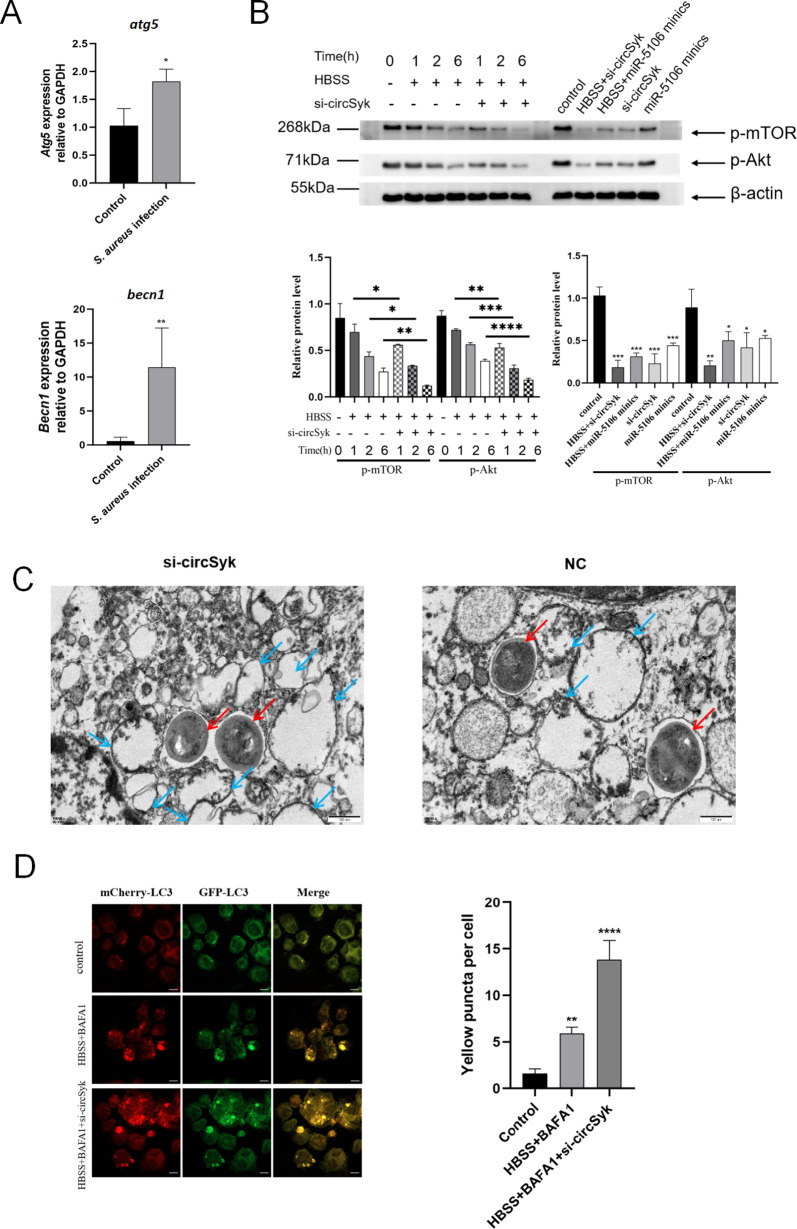
Impact of the circSyk and miR-5106 on autophagy in osteoclasts. (A) Expression of autophagy-related genes (*Atg5* and *Becn1*) determined with RT-qPCR in *S. aureus* infected-osteoclasts. (B) Osteoclasts were treated with HBSS with or without si-circSyk for 0, 1, 2, 6 h; treated with the transfection of siRNA-circSyk and miR-5106 minics with or without HBSS treament, and subjected to western blot analysis of p-mTOR and p-Akt. (C) Autophagosomes in *S. aureus*-infected osteoclasts of si-circSyk and NC group were observed by transmission electron microscopy. The blue arrow points to autophagosomes. The red arrow points to *S. aureus*. Scale bar: 500 nm. (D) Detection and quantification of autophagic flux in *S. aureus*-infected osteoclasts with the mCherry-GFP-LC3B reporter in control, HBSS+BAF-A1, and HBSS+BAF-A1+si-circSyk groups. Yellow puncta, autophagosomes (mCherry^+^/GFP^+^); red puncta, autolysosomes (mCherry^+^/GFP^−^). Scale bar: 10 μm. Data are means ± SD of three independent experiments (*P < 0.05; **P < 0.01; ***P < 0.001; ****P<0.0001; ns, nonsignificant).

As shown in Fig 5A, the expression of autophagy-related genes “*atg5*” and “*becn1*” were significantly upregulated in osteoclasts infected with *S. aureus*. Considering that numerous studies reported the association of Sik3 with the classic autophagy pathway Akt-mTOR [39,40], we detected the levels of phosphorylated mTOR (p-mTOR) and phosphorylated Akt (p-Akt). After osteoclasts were treated by HBSS starvation, a time-dependent downregulation of phosphorylated mTOR and Akt was observed, and silencing circSyk further reduced the levels of phosphorylated mTOR and Akt (Fig 5B). Additionally, osteoclasts treated with si-circSyk or miR-5106 mimics alone showed reduced phosphorylation of mTOR and Akt, while the combination treatment with HBSS further enhanced this reduced effect (Fig 5B). These results suggest that circSyk may inhibit the autophagy of osteoclasts through the classic Akt/mTOR-dependent signaling pathway. As shown in Fig 5C, more autophagosomes were observed in the si-circSyk group compared with the NC group through transmission electron microscope analysis, indicating a higher level of autophagy in *S. aureus*-infected osteoclasts in which circSyk was silenced. We utilized the mCherry-GFP-LC3 reporter to assess and quantify autophagy flux in *S. aureus*-infected osteoclasts under various treatment conditions. As shown in Fig 5D, the number of autophagosomes in *S. aureus*-infected osteoclasts treated with HBSS and BAF A1 increased significantly compared to that of control group, and silencing circSyk further enhanced the accumulation of autophagosomes (Fig 5D). Moreover, BAF A1 inhibited the fusion of autophagosomes with lysosomes, resulting in fewer free red puncta in the BAF A1-treated groups (Fig 5D).

**Fig 6:**
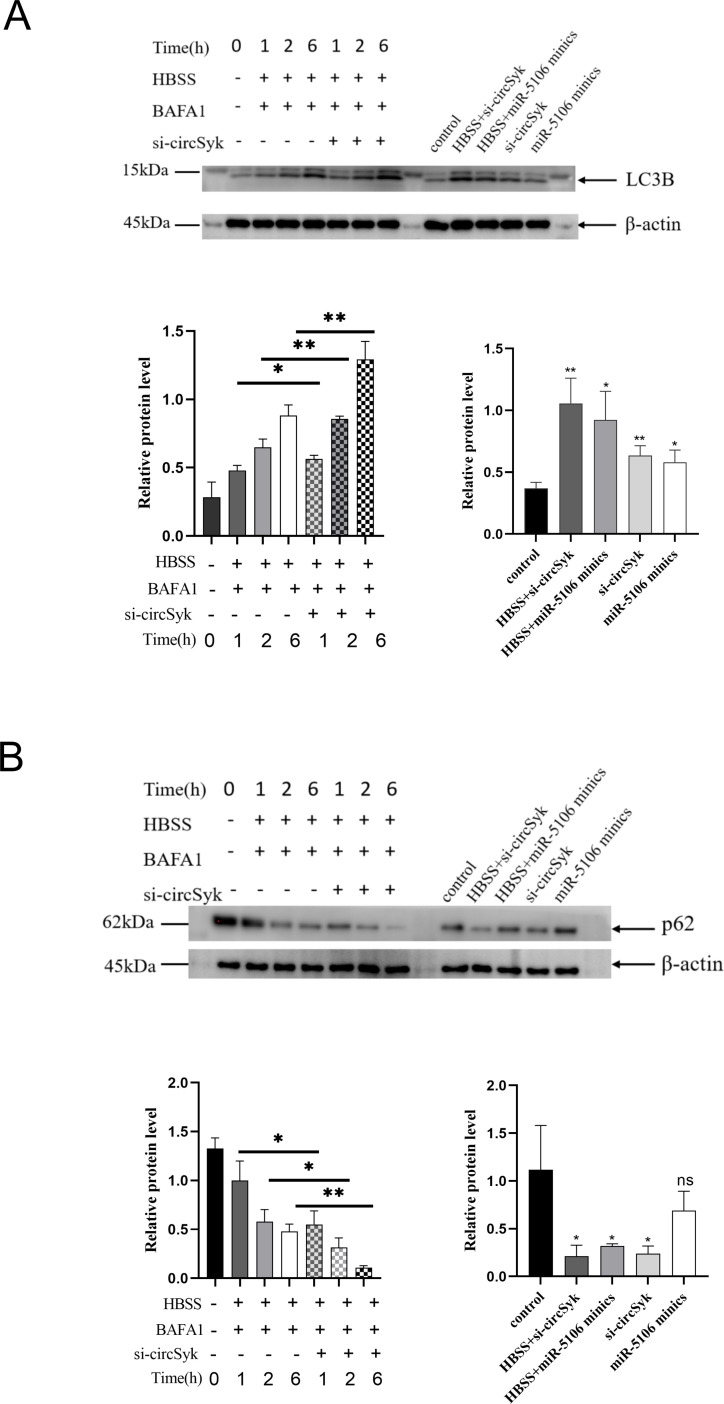
Analysis of autophagic flux in osteoclasts. Osteoclasts were exposed to HBSS and BAF-A1 with or without si-circSyk for 0, 1, 2 and 6 h; treated with the transfection of siRNA-circSyk and miR-5106 minics with or without HBSS treament, and subjected to western blot analysis of LC3B (A) and p62 (B). Data are means ± SD of three independent experiments (*P < 0.05; **P < 0.01; ns, nonsignificant).

It was reported that autophagic flux is determined over time and can provide insights into the dynamics of autophagy regulation [41]. The expression of the autophagy markers LC3B and p62 was investigated due to previous reports indicating that more active autophagy involves lower p62 and higher LC3B expression [42–44]. As shown in Fig 6A, the expression of LC3B was significantly upregulated in a time-dependent manner in HBSS and BAF A1 co-treated *S. aureus*-infected osteoclasts, and silencing circSyk further enhanced LC3B expression on the basis of HBSS and BAF A1 treatment, while the expression of p62 showed an opposite trend compared to LC3B in the same condition (Fig 6B). Additionally, treatment with si-circSyk or miR-5106 mimics individually elevated LC3B expression and reduced p62 levels in osteoclasts, and this effect was further intensified when combined with HBSS-induced starvation (Fig 6).

**Fig 7:**
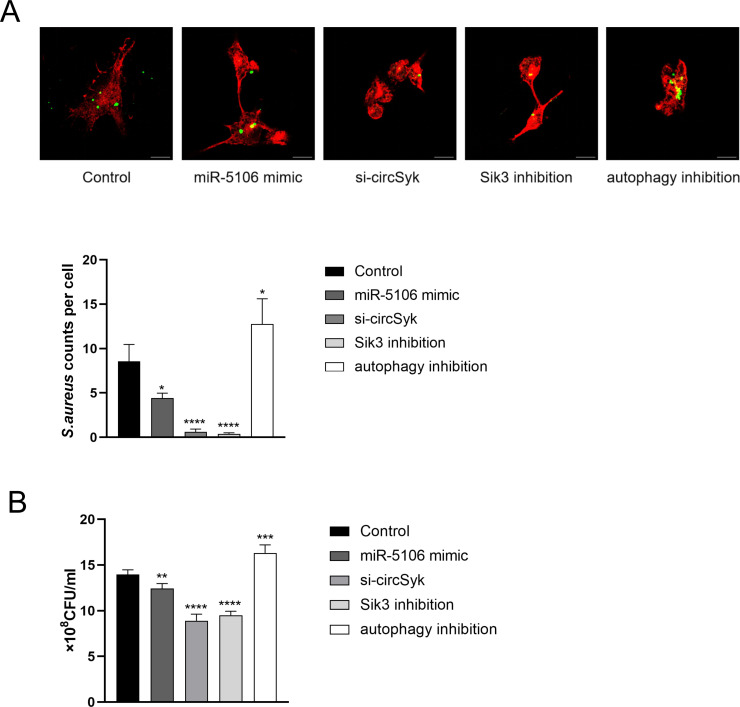
Impact of the circSyk/miR-5106/Sik3 axis on intracellular survival of *S. aureus* in osteoclasts. Immunofluorescence assays (A) and plate dilution assays (B) of the intracellular survival of *S. aureus* within osteoclasts were performed. Osteoclasts were divided into the following treatment groups: Control, si-circSyk, miR-5106 mimic, autophagy inhibition (BAF-A1 treatment), and Sik3 inhibition. Data are means ± SD of three independent experiments (*P < 0.05; **P < 0.01; ***P < 0.001; ****P < 0.0001).

Bacterial infection can induce autophagy in the host [45,46]. Vozza *et al*. discussed the role of autophagy in the intracellular survival of *S. aureus* within neutrophils [47]. To determine whether circSyk/miR-5106/Sik3 axis had a direct effect on the intracellular survival of *S. aureus*, we utilized immunofluorescence assays to investigate the intracellular survival of *S. aureus* within osteoclasts. As shown in Fig 7A, the intracellular survival of *S. aureus* was significantly decreased in the si-circSyk, miR-5106 mimic, and Sik3 inhibition groups compared to the control group. Conversely, we observed a significant increase in intracellular survival in the autophagy inhibition group (Fig 7A). The results obtained from the dilution plating method are consistent with the previously mentioned findings (Fig 7B).

### Verification of the circSyk/miR-5106/Sik3 axis in a mouse model

**Fig 8:**
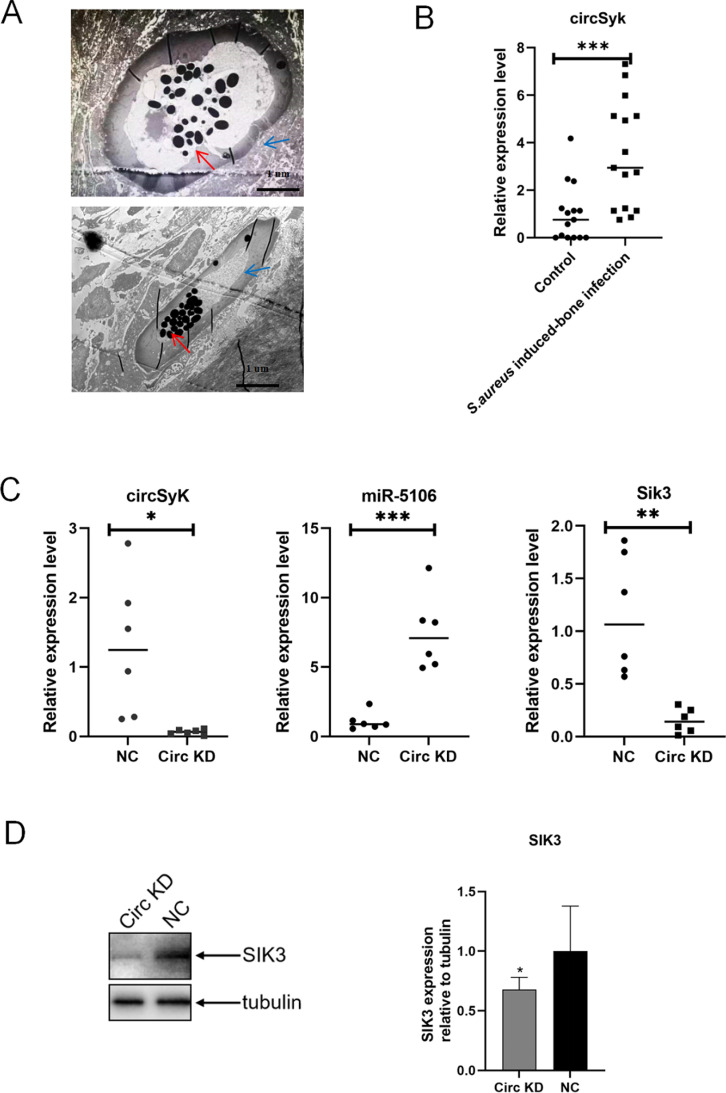
Verification of the circSyk/miR-5106/Sik3 axis in the mouse model. (A) Bone sample from a mouse model of *S. aureus*-infected bone was observed with transmission electron microscopy. *S. aureus* colonization was detected in bone tissue. Blue arrows indicate the hole in bone tissue formed by *S. aureus* invasion. Red arrow indicates the colonization of bone tissue by *S. aureus*. (B) CircSyk expression in mice with *S. aureus*-infected bone. Mice implanted with sterile pins were the control group.(n=15) (C) Expression levels of circSyk, miR-5106, and *Sik3* from adenovirus expressing siRNA-circSyk, which was injected into mice suffering *S. aureus*-infected bone, determined with RT-qPCR. Mice with *S. aureus*-infected bone and injected with adenovirus expressing siRNA-NC were the control group. (D) Sik3 expression in mice with *S. aureus*-infected bone and injected with circSyk-siRNA was determined with western blotting. Mice with *S. aureus*-infected bone and injected with adenovirus expressing siRNA-NC were the control group. Data are means ± SD of six independent experiments per group. (*P < 0.05; **P < 0.01; ***P < 0.001).

The mouse model of *S. aureus*-induced bone infection was confirmed by observing *S. aureus* colonization of bone tissue with transmission electron microscopy (Fig 8A). The serum levels of circSyk were analyzed with RT-qPCR. At 12 days post-inoculation, circSyk expression was significantly upregulated (Fig 8B). Adenovirus containing circSyk siRNA was injected into the mice and the knockdown efficiency was confirmed. As shown in Fig 8C, the levels of circSyk and *Sik3* expression were significantly downregulated, while miR-5106 expression was increased in the circSyk-knockdown (circ KD) group. In addition, Sik3 protein was significantly decreased in the circ KD group (Fig 8D).

### circSyk inhibition significantly reduced bacterial load and cortical bone destruction by up-regulating host autophagy

**Fig 9:**
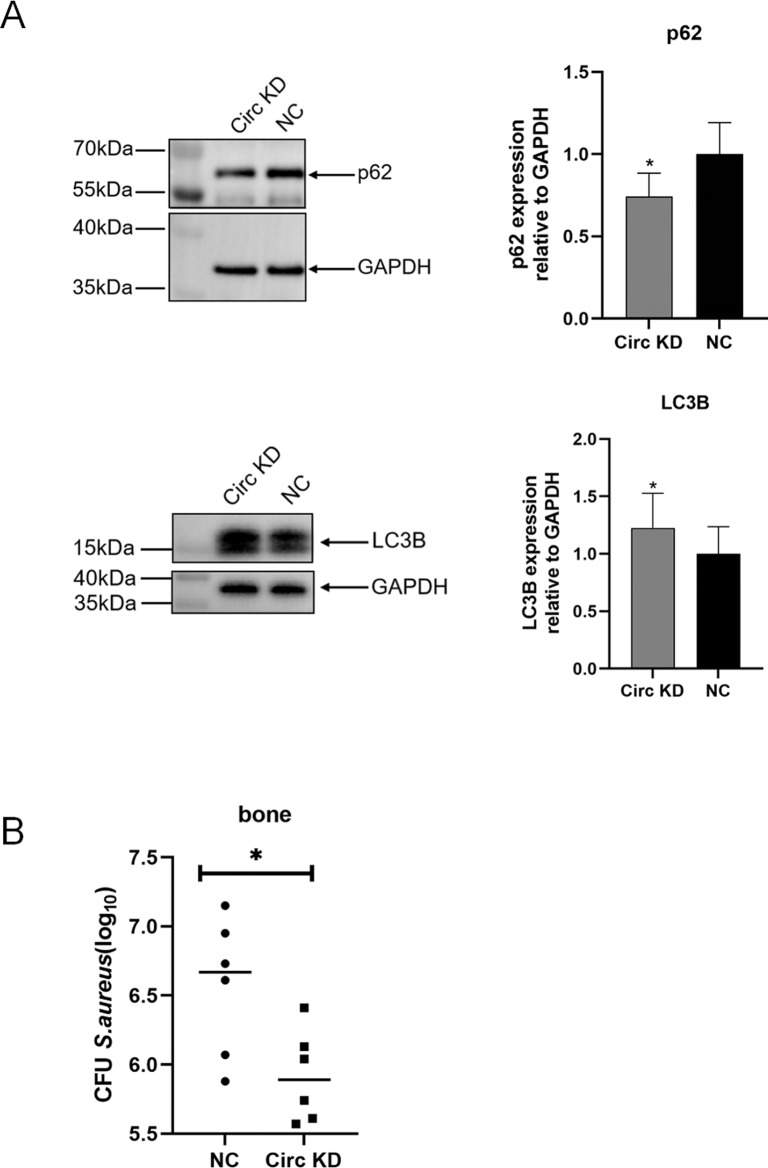
circSyk inhibition significantly reduces bacterial load in *S. aureus*-infected bone. (A) P62 and LC3B expression levels after siRNA-circSyk injection in mice with *S. aureus*-infected bone, determined with western blotting. (B) Bacterial load in bone tissue of mice with *S. aureus*-infected bone. Mice with *S. aureus*-infected bone and injected with adenovirus expressing siRNA-NC were the control group, called NC group. Data are means ± SD of six independent experiments per group (*P < 0.05).

The level of autophagy was determined in the mouse model through investigating the expression of autophagy marker p62 and LC3B. Mice with *S. aureus*-infected bone and injected with adenovirus expressing siRNA-NC served as the control group. And mice with *S. aureus*-infected bone and injected with adenovirus expressing siRNA-circSyk were designated as the Circ KD group. As shown in Fig 9A, the knockdown of circSyk expression (Circ KD group) significantly decreased p62 and elevated LC3B protein levels respectively, which up-regulated the host autophagy, indicating that the high expression of circSyk inhibited host autophagy in *S. aureus*-infected bone *in vivo*.

To assess the effects of circSyk on the bacterial load during bone infection, we quantified *S. aureus* CFU *in vivo*. A smaller bacterial load was observed in the bone tissue of Circ KD group mice than in that of the NC group mice (Fig 9B).

**Fig 10:**
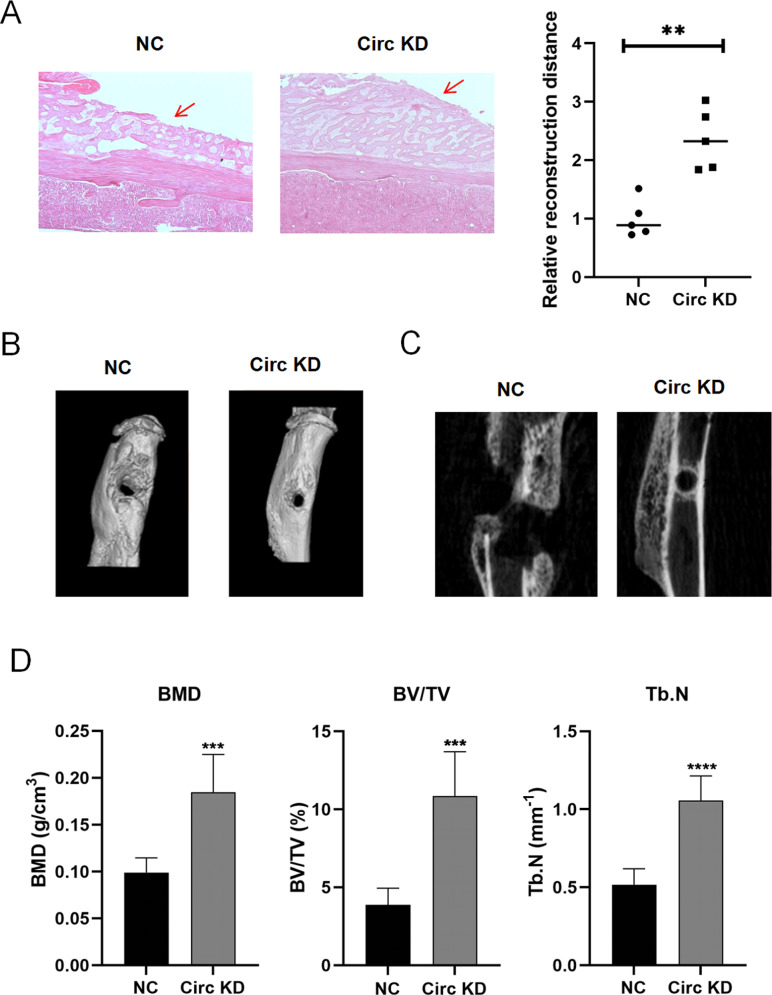
circSyk inhibition significantly reduces bone destruction in *S. aureus*-infected bone. (A) H&E staining of *S. aureus*-infected bone tissue from mice. Red arrow indicates the areas of bone repair. (B) Three-dimensional reconstruction of microCT image of tibiae from mice with *S. aureus*-infected bone in the circSykKD and NC groups. (C) MicroCT images of tibial sections from mice with *S. aureus*-infected bone in the circSyk KD and NC groups. Mice with *S. aureus*-infected bone and injected with adenovirus expressing siRNA-NC were the control group. (D) Tb.N, BMD, and BV/TV differed significantly between the circSyk KD and NC groups. Mice with *S. aureus*-infected bone and injected with adenovirus expressing siRNA-NC were the control, called NC group. Data are means ± SD of five independent experiments per group. (**P < 0.01; ***P < 0.001; ****<0.0001).

To investigate the effects of circSyk on bone resorption, H&E staining and microCT were used to assess the bone structure in the Circ KD and NC group mice which was injected with adenovirus expressing siRNA-circSyk and siRNA-NC, respectively. As shown in Fig 10A, mice in the Circ KD group showed better bone repair than those in the NC group. A microCT analysis of the tibiae identified differences between the Circ KD group and the NC group (Fig 10B and 10C). Significant differences in BMD, BV/TV, and Tb.N were observed between the Circ KD and NC groups, with the Circ KD group showing higher values for these indicators compared to the NC group (Fig 10D). This suggests more active trabecular bone remodeling and improved bone density in the Circ KD group, collectively indicating superior bone repair outcomes.

All these results confirmed that in mice with *S. aureus*-infected bone, the lower circSyk expression induced more active autophagy, smaller organ bacterial loads, decreased bone destruction and better bone repair. Therefore, circSyk is a compelling target in the treatment of bone infection caused by *S.aureus*.

## Discussion

Circular RNAs have long been recognized as the products of abnormal gene splicing and were thought to have no function [48]. However, several studies have shown that they regulate both pathological and physiological processes in multiple ways, particularly during bacterial infection [49,50]. Liu *et al.* demonstrated the importance of circTmem241 in innate lymphoid cells, in which it interacts with NONO protein to recruit the histone methyltransferase ASH1L onto the *Elk3* promoter in innate lymphoid progenitor cells and regulates the host immune response during bacterial infection [51]. Liu *et al.* also reported that the deletion of circZbtb20 reduced the number of group 3 innate lymphoid cells, increasing the mouse’s susceptibility to *Citrobacter rodentium* infection via the ALKBH5-dependent RNA m(6)A demethylation of *Nr4a1* mRNA [52]. Although circRNA plays an important role in the host immune response to bacterial infection, its role in *S. aureus*-infected bone remains unclear. Until now, research into bone infection has mainly focused on the impact of *S. aureus* on osteoblasts. Although the role of osteoclasts in this pathological process has received insufficient attention, several studies have shown that *S. aureus* can proliferate in osteoclasts, enhancing osteoclast maturation and bone resorption [31,53].

Based on our previous reports of the circRNA expression profile of osteoclast infected by *S. aureus* [29]. circSyk showed significantly elevated expression in *S. aureus-*infected osteoclasts in the present study. In addition, the expression of circSyk did not change in *Staphylococcus epidermidis-*infected osteoclasts (S1B Fig). Moreover, we confirmed the high levels of circSyk in the sera from patients with *S. aureus*-infected bone. We proposed that circSyk may be a potential novel biomarker for the diagnosis of *S. aureus*-induced bone infection although more clinical samples need to be verified for the specificity of circSyk, for instance, in other pathogens-induced bone infection.

In this study, we confirmed that *S. aureus*, a facultative intracellular pathogen, likely regulates its intracellular survival via upregulating the expression of circSyk, which is derived from the Syk gene. The Syk gene, as an essential player in immune cell signaling and osteoclastogenesis [54], has been shown to regulate immune responses [55], osteoclast differentiation, and bone resorption in various inflammatory and bone diseases [56]. Therefore, we hypothesize that *S. aureus* might modulate its intracellular survival in osteoclasts to promote progression of bone infection via these pathways associated with circSyk.

The ceRNA mechanism, also called the ‘sponge mechanism’, is composed of circRNA/miRNA/mRNA axis, and is involved in many biological processes [19–21]. In this study, we proposed that the circSyk/miR-5106/Sik3 axis may be a potential immune evasion pathway by which *S. aureus* blocks osteoclast autophagy to ensure its own survival. Sik3 is a serine/threonine kinase involved in the regulation of various cellular processes, including metabolism, inflammation, and immune responses [57]. For instance, Sik3 can influence skeletal development and cellular metabolism by altering the mTOR signaling pathway [39]. Given that mTOR is a well-known negative regulator of autophagy [58], this study suggested that Sik3 may modulate autophagy through its regulatory effects on the mTOR pathway and thereby intervene *S.aureus* evasion of the host immune response. Notably, Sik3 was elevated obviously during *S. aureus-*induced bone infection in this study, which may be developed for an ideal therapeutic target in the clinical treatment of bone infection, but it needs to be verified in the future. Moreover, circSyk affects bone repair through Sik3 *in vivo* in this study, suggesting that Sik3 not only contributes to the immune evasion of *S. aureus* but also influences the bone remodeling process. It is noteworthy that siRNA targeting circSyk significantly affected the outcome of bone infection. These results undoubtedly extend our understanding of the immune escape of *S. aureus* during bone infection.

Circular RNAs have become key regulators of various physiological processes, including bone metabolism and immune response regulation. Over the past decade, numerous studies have highlighted the important roles of circRNAs in bone-related diseases, though their precise mechanisms still need to be further clarified. Bone metabolism is a dynamic process involving the regulation of osteoblasts and osteoclasts, and circRNAs have been shown to influence both processes. Zhang *et al.* showed that in bone metabolism, circRNA-vgll3 regulates ITGA5 expression through miR-326-5p thereby affecting the osteogenic differentiation of adipose-derived mesenchymal stem cells [59]. Wang *et al.* proposed that circ_0008542 upregulates the expression of the *TNFRSF11A* gene by acting as a sponge for miR-185-5p. Furthermore, its 1916–1992 bp segment showed increased m6A methylation levels, which inhibited the RNA methyltransferase METTL3, while the overexpression of the RNA demethylase ALKBH5 reversed osteoclast differentiation and bone resorption [60]. In addition to their roles in bone metabolism, circRNAs have gained attention for their involvement in immune regulation. Xue *et al.* identified 35,342 and 6146 DEcircRNAs in rheumatoid arthritis patients compared with healthy individuals and patients with osteoarthritis, respectively, which are associated with cellular protein metabolic processes, gene expression, and the immune system, among other functions [61]. Kou *et al.* identified 123 DEcircRNAs associated with ankylosing spondylitis, which were annotated to the biological processes of peptidyl-serine phosphorylation and the human immune system [62]. These findings suggest that circRNAs can modulate immune responses within the bone microenvironment, potentially influencing the progression of autoimmune bone diseases. The present study extends our understanding of circRNAs in bone immunity and metabolism. We have shown that *S. aureus* evades the immune system through circSyk, causing greater bone destruction. Considering the particularity of the bone immune environment, drug treatments targeting circSyk may provide an effective approach to treating bone infections.

Tam *et al.* reported that *S. aureus* produces an array of bicomponent pore-forming toxins that target and kill leukocytes, called ‘leukocidins’, and that blocking leukocidin-mediated immune evasion protects the host during *S. aureus* infection [63]. Pauli *et al.* proposed that *S. aureus* infection induces protein-A-mediated immune evasion to limit the host’s responses to other *S. aureus* virulence factors, which would result in chronic or recurrent disease [64]^.^ *S. aureus* also uses host biological processes to evade the host’s immune system. Thwaites *et al*. proposed that polymorphonuclear neutrophils are key to *S. aureus* infection, acting as an intracellular survival Trojan horse that spreads to other cells during infection [65]. This evidence was validated in both mouse and human primary neutrophils [66–68]. Osteoclasts are the main immune cells in the bone immune environment, similar to Kupffer cells in the liver [69]. Therefore, we believe that our study is of significant interest. We demonstrated that the immune evasion mechanism of *S. aureus* during bone infection is involved autophagy, allowing *S. aureus* to evade the host’s immune response by regulating circSyk. Therefore, we believe that the immune escape pathway of *S. aureus* in bone infection has been identified, and has good potential therapeutic utility.

Autophagy plays a complex role in *S. aureus* infection. LC3B is an important marker of autophagy and functions in autophagosome formation [70]. p62 is another key biomarker of autophagy function, and is used to monitor autophagic flux [71]. Previous studies have shown that the autophagy proteins p62 and ATG16L1 regulate autophagy to affect the host immune defenses and the damage caused by *S. aureus* [15,16]. However, autophagy is currently considered an important pathway by which *S. aureus* evades the host’s immunity. It has also been reported that *S. aureus* inhibits autophagy to maintain its intracellular infection [17,18]. Gibson JF *et al*. demonstrated that neutrophils provide protection for against intracellular infection by *S. aureus* through Lc3-associated phagocytosis [15]. In the present study, a novel immune evasion pathway used by *S. aureus* has been demonstrated, based on the autophagy of osteoclasts. The key factor, circSyk, affects the outcome of bone infection at the RNA level. We confirmed that *S. aureus* blocks autophagy of infected osteoclasts by upregulating circSyk. Furthermore, a microenvironment conducive to bone destruction is created by *S. aureus* through the circSyk/miR-5106/Sik3 axis. Progressive bone destruction requires extensive surgical intervention and long-term antibiotic therapy, and the development of urgently required alternative strategies is the current challenge posed by bone infection.

In this study, the body weight and bacterial loads of mice were analyzed to assess the host response to *S. aureus.* We note that some other researchers have used similar analyses in their studies [72–74]. However, we stress that these results only incompletely describe in the host–pathogen interactions operating in this context. Further, the *S. aureus* bone infection model in mice does not fully simulate bone infection in humans.

To the best of our knowledge, this study has identified a crucial circRNA, circSyk, which blocks host autophagy via the circSyk/miR-5106/Sik3 axis to promote the progression of *S. aureus*-caused bone infection. Most importantly, circSyk could be used as an ideal potential therapeutic target for *S. aureus*-infected bone. In the future, we can develop the treatments of inhibiting circSyk to control the progression of bone infection caused by *S. aureus*.

## Conclusions

**Fig 11:**
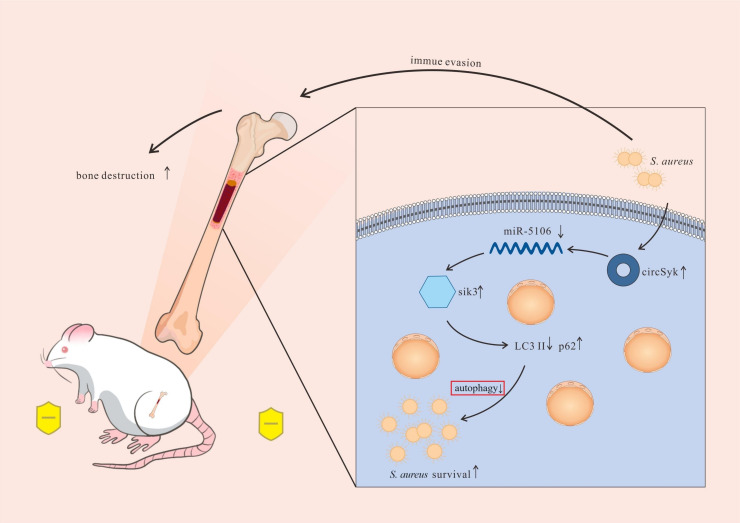
Model of *S. aureus* blocking autophagy to promote the progression of bone infection and bone destruction via the circSyk/miR-5106/Sik3 axis. Figure 11 was created by Figdraw. (www.figdraw.com).

This study has identified the important roles of circSyk in *S. aureus*-infected bone. Most importantly, a novel immune evasion pathway of *S. aureus* based on the autophagy of osteoclasts was demonstrated. During bone infection, *S. aureus* stimulates circSyk to block autophagy and promote bone destruction via the circSyk/miR-5106/Sik3 axis, as shown in Fig 11. Overall, we offer a new perspective for the pathogenesis of bone infection caused by *S. aureus*.

## Supporting information

S1 Fig (A) Expression levels of five additional miRNAs (miR-3064-5p, miR-6945-5p, miR-680, miR-6947-3p, and miR-744-3p) in S. aureus-infected osteoclasts. (B) Expression of circSyk in osteoclasts infected with *Staphylococcus epidermidis*. (C) Expression of miR-5106 and *Sik3* in osteoclasts with circSyk overexpression. OE-circSyk refers to circSyk overexpression. (D) Uncropped blots of Fig 8D. (E) Uncropped blots of Fig 9A. Data are means ± SD of five independent experiments per group. (*P < 0.05; **P < 0.01; ns, nonsignificant).
(TIF)

S2 Fig MicroCT images. (TIF)

S1 Table RNA sequences. (DOCX)

S2 Table qPCR primers. (DOCX)

S3 Table Clinical characteristics of the patients cohort. (DOCX)

S4 Table Potential downstream miRNAs of circSyk. (DOCX)

S5 Table Data for graph of this paper. (XLSX)
