# Supplementary figures and images for: Staphylococcus aureus blocks host autophagy through circSyk/miR-5106/Sik3 axis to promote progression of bone infection
Source: PLoS Pathog. 2025 Jan 27;21(1):e1012896. doi: 10.1371/journal.ppat.1012896 (PMC11781720; doi:10.1371/journal.ppat.1012896)

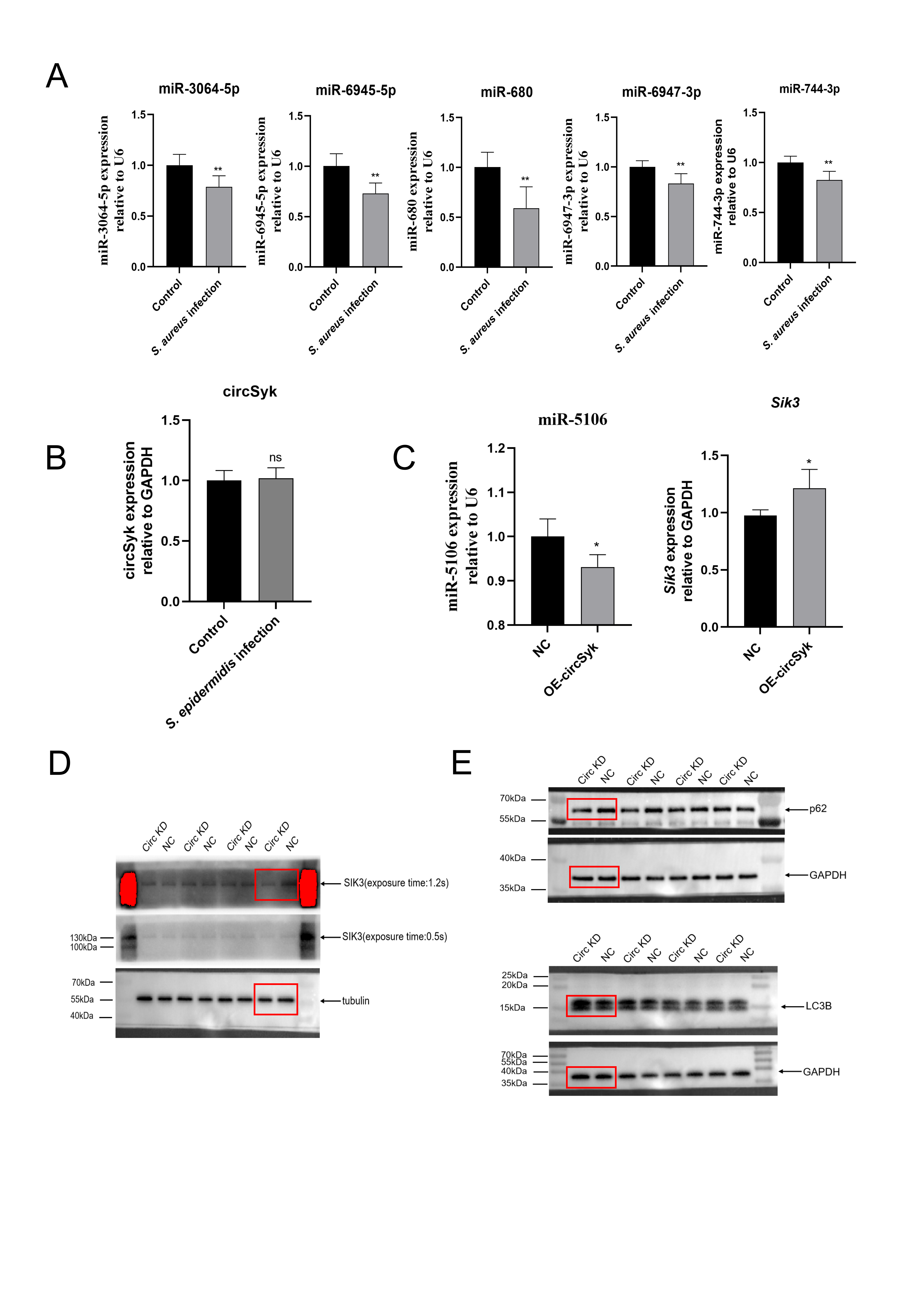

Supplement: S1 Fig — (A) Expression levels of five additional miRNAs (miR-3064-5p, miR-6945-5p, miR-680, miR-6947-3p, and miR-744-3p) in S. aureus-infected osteoclasts. (B) Expression of circSyk in osteoclasts infected with Staphylococcus epidermidis. (C) Expression of miR-5106 and Sik3 in osteoclasts with circSyk overexpression. OE-circSyk refers to circSyk overexpression. (D) Uncropped blots of Fig 8D. (E) Uncropped blots of Fig 9A. Data are means ± SD of five independent experiments per group. (*P < 0.05; **P < 0.01; ns, nonsignificant). (TIF) [file ppat.1012896.s001.tif]

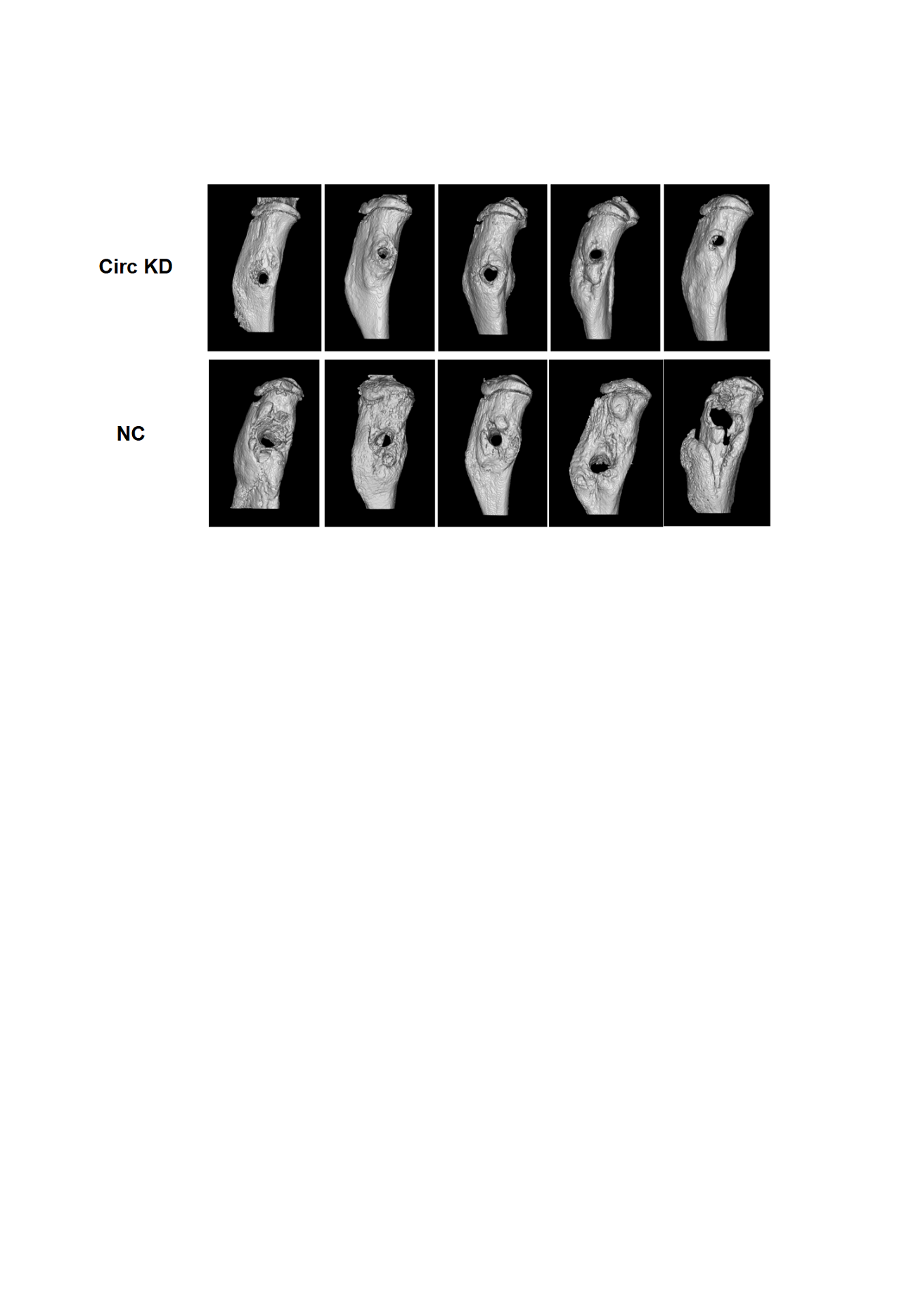

Supplement: S2 Fig — (TIF) [file ppat.1012896.s002.tif]
