# Supplementary material for: Staphylococcus aureus blocks host autophagy through circSyk/miR-5106/Sik3 axis to promote progression of bone infection
Source: PLoS Pathog. 2025 Jan 27;21(1):e1012896. doi: 10.1371/journal.ppat.1012896 (PMC11781720; doi:10.1371/journal.ppat.1012896)
Supplement: S1 Table — (DOCX) [file ppat.1012896.s003.docx]

| Table S1 Sequences | | |
| --- | --- | --- |
| **GeneName** | **Type** | **GeneSequence** |
| miR-5106 mimics | sense | AGGUCUGUAGCUCAGUUGGCAGA |
|  | antisense | UGCCAACUGAGCUACAGACCUUU |
| circSyk siRNA-1 | sense | GGAAAAUUCCUGUACUUCUTT |
|  | antisense | AGAAGUACAGGAAUUUUCCTT |
| circSyk siRNA-2 | sense | UUCCUGUACUUCUCCAUACTT |
|  | antisense | GUAUGGAGAAGUACAGGAATT |
| Negative Control (NC) | sense | UUCUCCGAACGUGUCACGUTT |
|  | antisense | ACGUGACACGUUCGGAGAATT |
| miR-5106-WT |  | CACCATCGAGAGGGAACTTAATGGCACCTACGCCATCTCCGGGGGCAGGGCCCATGCCAGCCCAGCAGACCTCTGCCATTACCACTCCCAGGAACCTGATGGCCTTATCTGCCTCCTTAAGAAGCCCTTCAACCGGCCCCCGGGAGTACAGCCCAAGACCGGACCCTTTGAGGACCTGAAGGAGAACCTCATCAGGGAATATGTGAAACAGACCTGGAACCTTCAGGGCCAGGCTCTGGAGCAAGCCATCATCAGCCAGAAGCCCCAGCTGGAGAAGCTGATCGCCA |
| miR-5106-MUT |  | CACCATCGAGAGGGAACTTAATGGCACCTACGCCATCTCCGGGGGCAGGGCCCATGCCAGCCCACTCTGGGACTGCCATTACCACTCCCAGGAACCTGATGGCCTTATCTGCCTCCTTAAGAAGCCCTTCAACCGGCCCCCGGGAGTACAGCCCAAGACCGGACCCTTTGAGGACCTGAAGGAGAACCTCATCAGGGAATATGTGAATGCTTGAGGGAACCTTCAGGGCCAGGCTCTGGAGCAAGCCATCATCAGCCAGAAGCCCCAGCTGGAGAAGCTGATCGCCA |
| *sik3* 3’UTR-WT |  | cccccccctctcgttttcacctaccatttctgaggtagatgaaccagaggttagtattcaggggctgaacctcaggaccctcccttccacagcctgtctcagaccagactctctccctctgccccggtctaccaggaacaaatgctagaccggagcttggtggccaagaaaaggagaggagggggcagtggcccctgcct |
| *sik3* 3’UTR-MUT |  | Cccccccctctcgttttcacctaccatttctgaggtagatgaaccagaggttagtattcaggggctgaacctcaggaccctcccttccacagcctgtcttgagttagactctctccctctgccccggtctaccaggaacaaatgctagaccggagcttggtggccaagaaaaggagaggagggggcagtggcccctgcct |
