# Supplementary material for: Staphylococcus aureus blocks host autophagy through circSyk/miR-5106/Sik3 axis to promote progression of bone infection
Source: PLoS Pathog. 2025 Jan 27;21(1):e1012896. doi: 10.1371/journal.ppat.1012896 (PMC11781720; doi:10.1371/journal.ppat.1012896)
Supplement: S2 Table — (DOCX) [file ppat.1012896.s004.docx]

| Table S2 qPCR primers | | |
| --- | --- | --- |
| **GeneName** | **PrimerType** | **GeneSequence** |
| mmu-circSyk | Forward | GAGTACAGCCCAAGACCGGA |
|  | Reverse | GTGGCGATCAGCTTCTCCAG |
| hum-circSyk | Forward | TCTCTCGGGAAGAATCTGAGCA |
|  | Reverse | AAAGAAGGGCAGGTGGTTGG |
| miR-6945-5p |  | ATACCATCCTGGGAGGGGCA |
|  |  |  |
| miR-744-3p |  | ACCTCATACTGTTGCCACTAACC |
|  |  |  |
| miR-3064-5p |  | AATCATGATCTGGCTGTTGTGGT |
|  |  |  |
| miR-680 |  | AAGTATTCGGGCATCTGCTGAC |
|  |  |  |
| miR-6947-3p |  | AGAGACGGCAGCCTCTTTCC |
|  |  |  |
| miR-5106 |  | AACCACTAGGTCTGTAGCTCAGT |
|  |  |  |
| Syne | Forward | GGCAACCTTGACCGAAATATAC |
|  | Reverse | GTAACATTTCTTGGGCATCGTC |
| Hbegf | Forward | CAAGCAAAGAAAGGAATGGGAA |
|  | Reverse | GATTCTCCACTGGTAGAGTCAG |
| Ago2 | Forward | TTCCGACACCTGAAGAACACATACG |
|  | Reverse | ACACGCTTGACTTCCGCATACAC |
| Emp2 | Forward | GACCAGCACAGATTCCCACTTCAG |
|  | Reverse | GGCAAGCAGAGACAACCCACAG |
| Sik3 | Forward | CTTGAACATGAATCGGTTCTCC |
|  | Reverse | CTGTAGTGCTGACGTGGTATAG |
| Glrx | Forward | CCTTTCAAACAAGGTCTTCTGG |
|  | Reverse | TGGAGATTAGATCACTGCATCC |
| mmu-U6 | Forward | CTCGCTTCGGCAGCACATATACT |
|  | Reverse | ACGCTTCACGAATTTGCGTGTC |
| mmu-GAPDH | Forward | AAGGTCATCCCAGAGCTGAA |
|  | Reverse | CTGCTTCACCACCTTCTTGA |
| hum-GAPDH | Forward | CAGGAGGCATTGCTGATGAT |
|  | Reverse | GAAGGCTGGGGCTCATTT |
