# Supplementary material for: Staphylococcus aureus blocks host autophagy through circSyk/miR-5106/Sik3 axis to promote progression of bone infection
Source: PLoS Pathog. 2025 Jan 27;21(1):e1012896. doi: 10.1371/journal.ppat.1012896 (PMC11781720; doi:10.1371/journal.ppat.1012896)
Supplement: S3 Table — (DOCX) [file ppat.1012896.s005.docx]

| **Table S3 Clinical characteristics of the patients cohort** | | |
| --- | --- | --- |
| Characteristic |  | Number (n =16) |
| Age |  |  |
|  | Median | 40 |
|  | Range | 21 to 78 |
| Gender | |  |
|  | Male | 11 |
|  | Female | 5 |
| Specimens | |  |
|  | Serum | 16 |
|  | Another | 0 |
| Comorbidities |  |  |
|  | Yes | 10 |
|  | No | 6 |
| Events |  |  |
|  | Novel | 3 |
|  | Relapse | 13 |
| Presence of implants |  |  |
|  | Yes | 11 |
|  | No | 5 |
| Experience of antibiotic treatment |  |  |
|  | Yes | 13 |
|  | No | 3 |
