# Supplementary material for: Staphylococcus aureus blocks host autophagy through circSyk/miR-5106/Sik3 axis to promote progression of bone infection
Source: PLoS Pathog. 2025 Jan 27;21(1):e1012896. doi: 10.1371/journal.ppat.1012896 (PMC11781720; doi:10.1371/journal.ppat.1012896)
Supplement: S4 Table — (DOCX) [file ppat.1012896.s006.docx]

| **Table S4 Downstream miRNA of circSyk** | |
| --- | --- |
| **Downstream miRNA** | **Energy** |
| mmu-miR-5106 | -28.10 |
| mmu-miR-6945-5p | -28.02 |
| mmu-miR-680 | -27.65 |
| mmu-miR-6947-3p | -26.65 |
| mmu-miR-3064-5p | -25.95 |
| mmu-miR-744-3p | -24.92 |
| mmu-miR-6379 | -24.66 |
| mmu-miR-3085-3p | -24.05 |
| mmu-miR-3474 | -23.89 |
| mmu-miR-762 | -23.77 |
| mmu-miR-3093-5p | -23.74 |
| mmu-miR-6940-5p | -23.64 |
| mmu-miR-709 | -23.61 |
| mmu-miR-1943-3p | -23.19 |
| mmu-miR-5621-3p | -22.97 |
| mmu-miR-3085-5p | -22.51 |
| mmu-miR-7648-3p | -22.02 |
| mmu-miR-7051-3p | -21.28 |
| mmu-miR-8120 | -21.00 |
| mmu-miR-3090-3p | -20.66 |
| mmu-miR-7094b-2-5p | -20.44 |
| mmu-miR-6909-3p | -20.08 |
| mmu-miR-3099-5p | -19.99 |
| mmu-miR-1264-5p | -19.31 |
| mmu-miR-6897-3p | -17.73 |
| mmu-miR-6346 | -17.54 |
| mmu-miR-7215-3p | -16.55 |
| mmu-miR-7003-3p | -16.53 |
| mmu-miR-6971-3p | -16.50 |
| mmu-miR-7083-3p | -16.36 |
| mmu-miR-6350 | -15.59 |
| mmu-miR-670-5p | -15.27 |
| mmu-miR-5114 | -14.52 |
| mmu-miR-6916-3p | -14.35 |
| mmu-miR-292b-3p | -14.20 |
